# MicroRNA Expression Profiling of the Porcine Developing Hypothalamus and Pituitary Tissue

**DOI:** 10.3390/ijms141020326

**Published:** 2013-10-14

**Authors:** Lifan Zhang, Zhaowei Cai, Shengjuan Wei, Huiyun Zhou, Hongmei Zhou, Xiaoling Jiang, Ningying Xu

**Affiliations:** 1 College of Animal Science, Zhejiang University, Hangzhou 310058, China; E-Mails: lfzhang777@163.com (L.Z.); zhouhuiyun1985@163.com (H.Z.); hmzhou@zju.edu.cn (H.Z.); xiaolingjiang@zju.edu.cn (X.J.); 2 Laboratory Animal Research Center, Zhejiang Chinese Medical University, Hangzhou 310053, China; E-Mail: czw1234@163.com; 3 Department of Animal Sciences, Washington State University, Pullman, WA 99164, USA; E-Mail: shengjuanwei@gmail.com

**Keywords:** miRNA, Solexa sequencing, microarray, hypothalamus, pituitary, pig

## Abstract

MicroRNAs (miRNAs), a class of small non-coding RNA molecules, play important roles in gene expressions at transcriptional and post-transcriptional stages in mammalian brain. So far, a growing number of porcine miRNAs and their function have been identified, but little is known regarding the porcine developing hypothalamus and pituitary. In the present study, Solexa sequencing analysis showed 14,129,397 yielded reads, 6,680,678 of which were related to 674 unique miRNAs. After a microarray assay, we detected 175 unique miRNAs in the hypothalamus, including 136 previously known miRNAs and 39 novel candidates, while a total of 140 miRNAs, including 104 known and 36 new candidate miRNAs, were discovered in pituitary. More importantly, 37 and 30 differentially expressed miRNAs from several developmental stages of hypothalamus and pituitary were revealed, respectively. The 37 differentially expressed miRNAs in hypothalamus represented 6 different expression patterns, while the 30 differentially expressed miRNAs in pituitary represented 7 different expression patterns. To clarify potential target genes and specific functions of these differentially expressed miRNAs in hypothalamus and pituitary, TargetScan and Gorilla prediction tools were then applied. The current functional analysis showed that the differentially expressed miRNAs in hypothalamus and pituitary shared many biological processes, with the main differences being found in tissue-specific processes including: CDP-diacylglycerol biosynthetic/metabolic process; phosphatidic acid biosynthetic/metabolic process; energy reserve metabolic process for hypothalamus; adult behavior; sterol transport/homeostasis; and cholesterol/reverse cholesterol transport for pituitary. Overall, this study identified miRNA profiles and differentially expressed miRNAs among various developmental stages in hypothalamus and pituitary and indicated miRNA profiles change with age and brain location, enhancing our knowledge about spatial and temporal expressions of miRNAs in the porcine developing brain.

## 1. Introduction

MicroRNAs (miRNAs) are a class of small non-coding RNA molecules, which function in transcriptional and post-transcriptional regulation of gene expression [1]. Since the first miRNA, lin-4 from *Caenorhabditis elegans*, was discovered in 1993 [2], more than 24,000 miRNAs from multiple species have been identified to date (miRBase 20.0). As important gene expression regulators, miRNAs have been demonstrated to be involved in many biological processes including cell differentiation, growth, apoptosis, and immune response [3,4]. Recently, with the development of small RNA sequencing technology, it is possible to identify miRNAs on a large-scale level in different species or tissues. Numerous studies for pig have been conducted on miRNA regulation and expression in the past three years. As a result, a large number of porcine miRNAs have been identified from different tissues, such as skeletal muscle, adipose tissue, cortex, cerebellum, ovary, and testis [5–9], providing increased evidence about the presence and functions of miRNAs in pig.

It is well known that the hypothalamic-pituitary-adrenal axis is a major part of the neuroendocrine system that regulates reactions of digestion, immune system, energy storage and expenditure [10]. In brief, the hypothalamus is a nervous tissue connected to the pituitary by nerve fibers and plays vital roles in nutritional intake and energy balance [11–13], while the pituitary works through regulating hormones and growth factors and is usually considered as a pea-sized endocrine gland located at the base of brain [14]. As such, miRNA profiling of hypothalamus and pituitary might provide novel clues for how miRNAs are involved in regulating the development of body growth and energy metabolism.

However, only one group, which investigated the miRNA profile on day 180 in Lantang pig [15], focused on miRNA identification in porcine pituitary tissue, and little is known about miRNA profiles in porcine hypothalamus. Therefore, we conducted miRNA expression profiling of porcine hypothalamus and pituitary by Selexa sequencing. Furthermore, a porcine miRNA array was designed according to our Solexa sequencing results and miRNA expressions in the developing hypothalamus and pituitary were analyzed using this array. The presence and identity of miRNAs could indicate post-transcriptional regulation of identified target genes involved in the regulation of growth and metabolism, providing a fundamental basis for experimental research.

## 2. Results

### 2.1. Solexa Sequencing Analysis of Small RNAs

**Figure 1:**
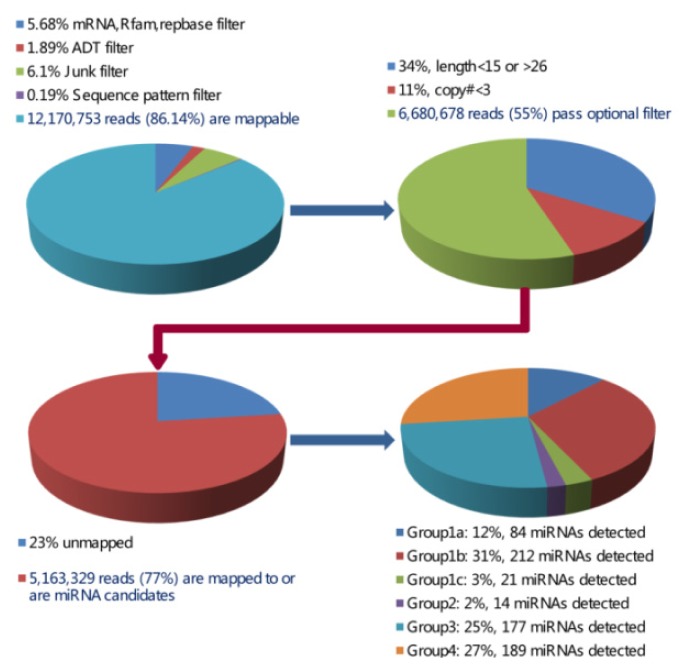
The chart of sequencing data analysis through various filters and the number of miRNAs detected.

A small RNA library was constructed using pooled RNA of hypothalamus and pituitary, followed by Solexa sequencing to discover new miRNA candidates. As shown in Figure 1, a total of 14,129,397 reads were obtained from our small RNA library. In addition, 6,646,023 reads with simple sequences, the length of sequences <15 nt or >26 nt, junk sequences, or sequences <3 copies were observed. After excluding the reads described above, the remaining 7,483,374 reads were compared with Hit mRNA, RFam and Repbase to remove possible mRNA, rRNA, tRNA, snRNA, snoRNA and repeat sequences. Finally, 6,680,678 reads with different length distribution (Figure S1) were held for miRNA identification.

After mapping to the genome of pig, a total of 674 unique miRNAs were identified (Figures 1 and S2) and divided into the following four groups: Group 1a, 1b, 1c; 2; 3; and 4 (Figure 1 and Table S1). Here group 1a and 1b were considered as the known miRNAs, while others were new candidates. Moreover, a similar naming method as used in Wei *et al.* [16] was applied for “PN” and “PC” groups (“PN” refers to sequences that have other mammalian homologous miRNAs, while “PC” shows sequences that only have *Sus scrofa* genomic cognates). A total of 380 miRNAs, including 214 in “PC” group and 166 in “PN” group, were identified further. More importantly, we compared our dataset to known pig miRNAs of miRBase 20.0 and other researchers, which were acquired by the technology of Solexa sequencing or LC Sciences [5,6,9,17–26]. Comparative results showed only 171 known miRNAs were mapped in the 380 miRNAs identified in this study (Table S1). Therefore, a total of 209 miRNAs were selected as new candidates.

### 2.2. Identification of Porcine miRNA Candidates by Microarray Assay

In order to confirm our new candidate miRNAs further, a microarray assay was performed using 819 miRNAs (Table S2). After discarding 644 candidates in hypothalamus and 679 candidates in pituitary (<100 average expressions, or signals >100 but had “bad spots”), the remaining 175 miRNAs in hypothalamus (Figure S3) and 140 miRNAs in pituitary (Figure S4) were used for further analysis. Combining the data from Solexa sequencing and miRNAs detected by microarray assay, 39 miRNAs in hypothalamus (including 17 conserved miRNAs with homologous miRNAs in other mammals and 22 novel candidates) and 36 miRNAs in pituitary (including 16 conserved miRNAs and 20 novel candidates), were identified by both technologies of Solexa sequencing and microarray assay (Table S1). As such, the data presented novel candidate miRNAs in pig. Interestingly, hypothalamus and pituitary shared 28 miRNAs including 11 conserved miRNAs and 17 novel candidates (Table S1), suggesting these miRNAs might be conserved between these two tissues.

### 2.3. Discovery of Differentially Expressed miRNAs during Hypothalamus and Pituitary Development

**Figure 2:**
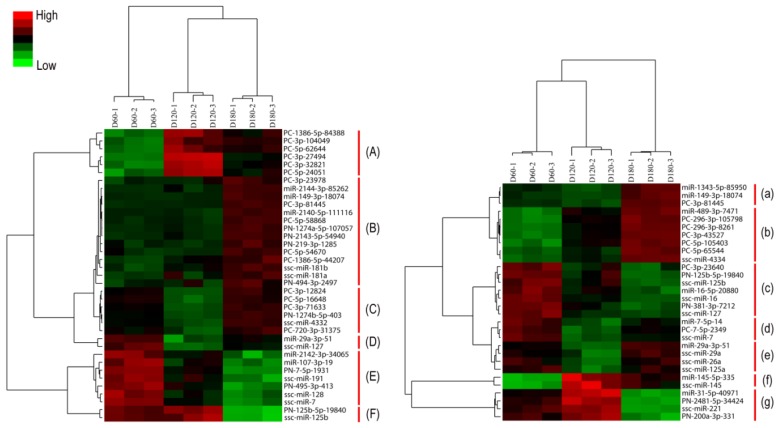
Differentially expressed miRNAs in the porcine hypothalamus and pituitary identified by SAM analysis. D60: day 60; D120: day 120; D180: day 180. A normalized and log-transformed data adjustment was performed in the cluster analysis of hypothalamus (**left**) and pituitary (**right**). The English letters represent different categories.

To identify miRNAs that might be involved in hypothalamus and pituitary development, we compared miRNA expression profiles of different developmental stages in hypothalamus and pituitary, respectively. Among the 175 and 140 identified miRNAs in hypothalamus and pituitary, 138 miRNAs in hypothalamus and 110 miRNAs in pituitary were discarded in further analysis as the CV of their biological repeats >0.3 or fold changes <2, *q* > 0.01, FDR > 0.001. Finally, 37 unique differentially expressed miRNAs in hypothalamus were identified among various developmental stages investigated (Figure 2) and 9 of these miRNAs changed more than ten-fold (Table 1). For example, miR-149-3p-18074 signals increased 12.20-fold and 8.18-fold from D120 to D180 and from D60 to D180, respectively; the increase of ssc-miR-4332 signals from D120 to D180 and from D60 to D180 were 42.20-fold and 8.81-fold respectively, with 4.76-fold decrease from D60 to D120; the decrease of ssc-miR-7 signals from D60 to D120 and from D60 to D180 were 14.29-fold and 12.50-fold, respectively.

Similarly, 30 unique differentially expressed miRNAs in pituitary were also identified (Figure 2) and 12 of these miRNAs changed more than ten-fold (Table 1). For example, PC-296-3p-8261 signals increased 11.22-fold and 12.57-fold from D120 to D180 and from D60 to D180, respectively; miR-1343-5p-85950 signals increased 14.10-fold and 3.73-fold from D120 to D180 and from D60 to D180, respectively, with 3.85-fold decrease from D60 to D120.

### 2.4. Expression Models of Differentially Expressed miRNAs

As shown in Figure 2 and S5,S6, miRNA expression patterns of hypothalamus and pituitary shared six typical categories: (A)/(f) expression level increased from D60 to D120/D180; (B)/(b) expression level increased from D60/D120 to D180; (C)/(a) expression level decreased from D60 to D120, but increased from D60/D120 to D180; (D)/(e) expression level decreased from D60/D180 to D120; (E)/(c) expression level decreased from D60 to D120/D180; (F)/(g) expression level decreased from D60/D120 to D180. Interestingly, one special expression mode was only found in pituitary as follows: (d) expression level decreased from D60 to D120/180, but increased from D120 to D180.

### 2.5. Prediction and Analysis of miRNA Target Genes

In this study, a total of 4191 and 4007 unique target genes from 37 and 30 differentially expressed miRNAs were successfully obtained, respectively (Tables S3 and S4). Unexpectedly, similar targets were predicted from miRNAs with high sequence similarity, *i.e.*, ssc-miR-7 and PN-7-5p-1931, PN-125b-5p-19840 and ssc-miR-125b in hypothalamus (Table S3); PC-296-3p-8261 and PC-296-3p-105798, ssc-miR-145 and miR-145-5p-335 in pituitary (Table S4). To define biological roles of all target genes, the gene ontology (GO) analysis was carried out further. As shown in Tables S5 and S6, 174 and 225 significant GO terms representing a wide range of biological processes were found for hypothalamus and pituitary respectively. Interestingly, the amounts of metabolic processes were the same in hypothalamus and pituitary, although only seven differentially expressed miRNAs were discovered in both tissues.

### 2.6. Assessment of miRNA Expression by Real-Time RT-PCR

**Figure 3:**
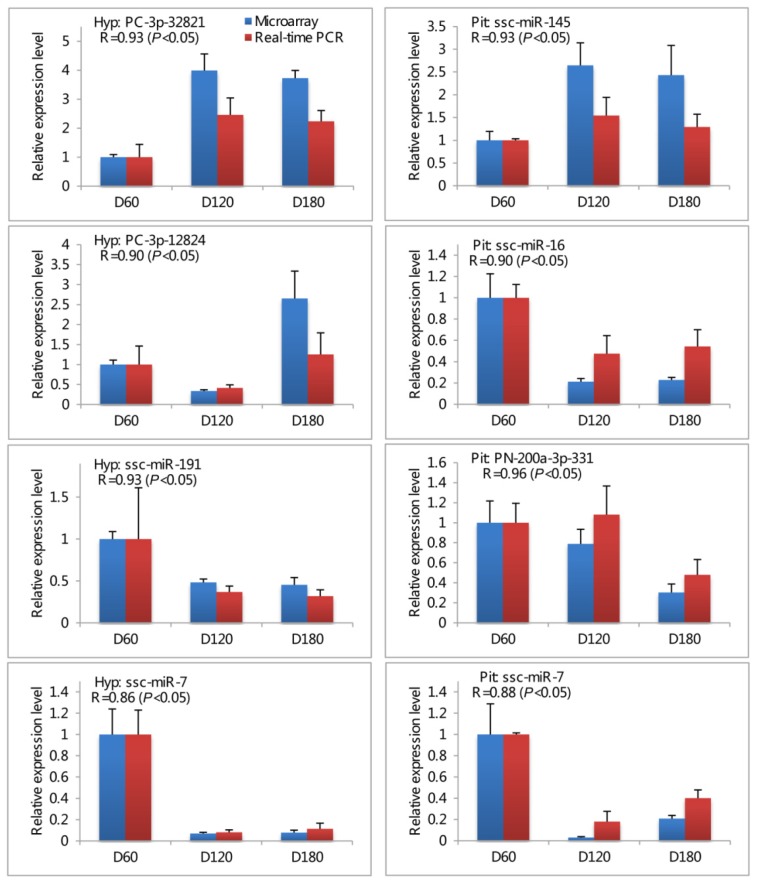
Validation of microarray results using real-time PCR method. Expression levels of seven miRNAs (PC-3p-32821, PC-3p-12824, and ssc-miR-191 for hypothalamus; ssc-miR-145, ssc-miR-16, and PN-200a-3p-331 for pituitary; ssc-miR-7 for both hypothalamus and pituitary) were detected by microarray (**left**) and real-time PCR (**right**). Hyp: hypothalamus; Pit: pituitary. All data were presented as the mean ± standard deviation (SD). Pearson correlation coefficient (*R*) was performed to determine the consistency between real-time PCR and microarray data. *p* value less than 0.05 was considered statistically significant correlation. Experiments were replicated three times using independent samples at each time point.

Seven differentially expressed miRNAs (PC-3p-32821, PC-3p-12824, and ssc-miR-191 for hypothalamus; ssc-miR-145, ssc-miR-16, and PN-200a-3p-331 for pituitary; ssc-miR-7 for both hypothalamus and pituitary) were chosen for validation by real-time RT-PCR using the same RNA samples that were applied to the microarray. The expression levels of all seven miRNAs determined by real-time PCR were concordant with their microarray data (Pearson correlation coefficient were 0.86–0.93 and 0.88–0.96 for hypothalamus and pituitary respectively; Figure 3), indicating the strong correlation between microarray profiling and the real-time RT-PCR data.

## 3. Discussion

In the present study, two powerful technologies, Solexa sequencing and microarray assay, were applied to acquire the miRNA expression profiles of porcine hypothalamus and pituitary in three developmental stages, and the stem-loop real-time RT-PCR was chosen to verify the data obtained by microarray assay. A total of 39 miRNAs in hypothalamus (including 17 conserved miRNAs and 22 new candidates) and 36 miRNAs in pituitary (including 16 conserved miRNAs and 20 new candidates) were successfully identified. Furthermore, 37 and 30 differentially expressed miRNAs with 4191 and 4007 potential target genes in hypothalamus and pituitary respectively were also found in our study. Our data revealed miRNA expression profiles of porcine developing hypothalamus and pituitary, providing new guidance for the expression of differentially expressed miRNAs.

As the most important local pig breed in China, Jinhua pig has higher backfat thickness, greater intramuscular fat content but lower growth rate compared to other commercial pig breeds such as Landrace [27]. In this study, three different developmental stages, D60, D120, and D180, were selected for the following reasons: D60 is the start time of rapid growth period in fattening pigs; D120 is almost the time of the growth peak in Jinhua pig [28]; D180 is the marketing time for Jinhua pig. Indeed, other studies in Jinhua pig also considered D120 and D180 as important developmental stages [29,30]. Therefore, investigations of miRNA expression patterns in these developmental time points may provide useful information about the miRNA temporal expressions in brain.

Most recently, Solexa sequencing technology has been considered as a maturing, convincing and effective strategy for identifying new miRNAs. To date, hundreds of potential porcine miRNAs have been successfully identified in porcine tissues by Solexa sequencing, *i.e.*, heart, liver, stomach, spleen, lung, kidney, small intestine, thymus, adipose tissue, muscle, ovary and testicle [5,6,9,18–24]. Li *et al.* [15] reported the miRNA profile of porcine pituitary in Lantang pig on D180 (another Chinese local pig breed) by Solexa sequencing, but data from D60 and D120 were not included. Here our study selected three time points (D60, D120, and D180) for investigating miRNA profiles of porcine developmental pituitary as well as hypothalamus by Solexa sequencing and microarray assay technologies. As a whole, 175 and 140 miRNAs in hypothalamus and pituitary respectively were identified in our study. Among all new candidate miRNAs, 17 of 39 and 16 of 36 miRNAs in hypothalamus and pituitary respectively were conserved miRNAs, which further supported the opinion that miRNA sequences were evolutionarily conserved [31]. On the other hand, we found many non-conserved new candidates (22 miRNAs in hypothalamus and 20 miRNAs in pituitary), indicating these candidates might be specific candidates in pig and have special functions in hypothalamus and pituitary tissues. However, additional studies are still required to confirm their specific roles in porcine hypothalamus and pituitary.

Generally speaking, the hypothalamus is defined as a key integrator of peripheral signals related with energy storage and nutrient status [32]. Evidence from studies with chicken and mouse indicate that miRNAs play a key role in the hypothalamus; high expression levels of miR-7 and miR-7b were discovered in mouse hypothalamus [33] and target genes from 12 of 179 differentially expressed miRNAs between 1-day and 36-week were associated with metabolism and development in chicken hypothalamus tissue [34]. However, little is known about profiles of porcine hypothalamus miRNAs. Our data showed that ssc-miR-7 or the highly similar sequence PN-7-5p-1931 decreased from D60 to D120/D180 (Figure 2 and Table 1), demonstrating that miR-7 might also be involved in the development of porcine hypothalamus. Interestingly, the maximum differentially expressed model of miRNAs is the increase of expression levels from D60/D120 to D180 (Figure 2 and Table 1), suggesting the altered miRNA expressions might be related to the late stage of Jinhua pig development.

As a critical endocrine organ, the pituitary produces hormones that influence body growth and bone metabolism. Previously, a number of studies revealed that miRNAs had important roles in the pituitary of human, mouse and pig: mir-7 and mir-7b were abundant in mouse pituitary [33]; miR-16 were expressed at lower levels in human pituitary adenomas as compared to normal pituitary tissue [35]; miR-26a played an important role in cell cycle control of ACTH-secreting human pituitary adenomas by modulating protein kinase Cδ [36]; miR-145 was found to be down-regulated in ACTH-secreting pituitary tumors [37]; miR-7, miR-125a/b, miR-26a and miR-29a had strong signals on D180 in porcine pituitary, while miR-221, miR-489, miR-145 and miR-16 possessed low expression signals, with moderate signals for miR-127 and miR-149 [15]. Our results also revealed these above miRNAs were differentially expressed, and provided further evidence for interpreting their roles in the pituitary. On the other hand, some miRNAs with unknown functions (e.g., miR-4334 and miR-1343) and other new differentially expressed candidates in porcine pituitary (e.g., PC-3p-81445, PC-3p-23640, PC-3p-43527, PC-5p-105403, PC-5p-65544, PN-200a-3p-331, and PN-2138-5p-105096) were identified in our study, providing a new clue for their biological functions. Interestingly, one special mode, which referred to the decrease from D60 to D120/180 with the increase from D120 to D180, was only discovered in pituitary. The reverse change of this mode was in accordance with the tendency of body growth, which showed fattening pigs kept growing from D60 to D180, with growth peak appeared on D120. Also, we noticed that sequences of miRNAs in this mode were highly similar to miR-7, which was the most highly expressed miRNA in porcine and mouse pituitary [15,33]. Therefore, our results suggested miR-7 might be involved in body growth by acting on the pituitary in pig.

Importantly, gene ontology analysis of potential target genes from differentially expressed miRNAs discovered terms were involved in various biological processes (Tables S5 and S6). Although only seven differentially expressed miRNAs were discovered in both hypothalamus and pituitary, the targets of differentially expressed miRNAs in both tissues shared more than half of significant GO terms (Tables S5 and S6), such as fatty acid beta-oxidation, cellular iron ion homeostasis, metal ion homeostasis, triglyceride metabolic process, small GTPase mediated signal transduction, and skeletal muscle thin filament assembly, indicating the regulation of miRNAs in both tissues might partly share similar roles. On the other hand, the tissue-specific significant GO terms for hypothalamus and pituitary were also discovered respectively, *i.e.*, CDP-diacylglycerol biosynthetic/metabolic process, phosphatidic acid biosynthetic/metabolic process and energy reserve metabolic process for hypothalamus (Table S5); adult behavior, axon development/regeneration, lipoprotein metabolic process, cholesterol/reverse cholesterol transport and sterol transport/homeostasis for pituitary (Table S6). Collectively, this data provides a good basis for further research in deciphering the roles of miRNAs in hypothalamus and pituitary.

## 4. Experimental Section

### 4.1. Ethics Statement and Sample Collection

Nine Jinhua boars in this experiment, including three boars per time point on day 60 (D60), 120 (D120) and 180 (D180), were obtained from Jinhua pig breeding CO., LTD in Zhejiang, China. Experiments were performed according to Regulations for the Administration of Affairs Concerning Experimental Animals (Ministry of Science and Technology, China, revised in June 2004). Animal Care and Use Committee (IACUC) in Zhejiang University specifically approved this study. Animals were allowed access to feed and water *ad libitum* under the same normal conditions and were humanely sacrificed as necessary to ameliorate suffering. Eighteen samples, including three independent samples per tissue and per time point, were derived from hypothalamus and pituitary tissue on D60, D120, and D180. All tissue samples were immediately placed in liquid nitrogen and then stored at −80 °C.

### 4.2. RNA Isolation and Solexa Sequencing

Total RNA was extracted using a mirVana miRNA Isolation Kit (Ambion, Austin, TX, USA) according to the manufacturer’s protocol. Subsequently, the RNA samples, derived from both hypothalamus and pituitary tissues, were pooled with equal quantities for small RNA sequencing. Approximately 60 mg of small RNA from RNA pool was used for library preparation and sequencing and the library was sequenced on the Genome Analyzer GA-I (Illumina, San Diego, CA, USA) following the vendor’s recommended protocol for small RNA sequencing.

### 4.3. Data Processing

Sequence-seqs were processed using Illumina’s Genome Analyzer Pipeline software and then subjected to a series of data filtration steps, from the statistics of mammalian miRNAs in miRBase 16.0, to obtain mapping sequences using the ACGT101-miR program (LC Sciences, Houston, TX, USA).

### 4.4. μ-Paraflo MicroRNA Microarray Assay

A total of 819 miRNAs, including 583 miRNAs from our small RNA sequencing results, 145 miRNAs from miRBase 16.0, and 91 miRNAs from both two libraries, were used (Table S2). RNA samples in hypothalamus and pituitary tissues were also used in microarray assay, which was performed using a service provider (LC Sciences, Houston, TX, USA). Details of the microarray assay were described as Marsh *et al.* [38] and Li *et al.* [39].

Data was analyzed by first subtracting the background and then normalizing the signals using a LOWESS filter (Locally-weighted Regression) [40]. To be listed as detectable, a transcript must meet at least three conditions: signal intensity higher than 3× (background standard deviation), spot coefficient of variation (CV) < 0.5, and average expression signal > 100. Differentially expressed miRNAs were defined as fold changes > 2, *q* < 0.01, and FDR < 0.001 (using SAM software, version 4.0, Stanford University, Stanford, CA, USA, 2001) [41] and the CV of biological repeats in any stage <0.3 [42]. Data classification involved a hierarchical clustering method with Cluster 3.0 using average linkage and Euclidean distance metric, and was visualized with Java Treeview (version 1.1.6r2, Stanford University, Stanford, CA, USA, 2004).

### 4.5. Stem-Loop Real-Time RT-PCR

A miRNA quantification method was performed as described by Huang *et al.* [43]. Seven miRNAs from hypothalamus and pituitary, which were shown in Table S7, were analyzed. Eighteen independent hypothalamus and pituitary samples among three time points were used in real-time RT-PCR. Firstly, 1 mg total RNA was reverse transcribed using the 200 U M-MLV reverse transcriptase (Takara: 02640A, Takara, Dalian, China) and 1 mL Stem-loop RT primer in an Applied Biosystems 9700 Thermocycler with incubation at 30 °C for 15 min, 42 °C for 60 min and 85 °C for 5 min. Then, real-time PCR was performed using a standard SYBR Green PCR kit (Takara: DRR041A, Takara, Dalian, China) and the StepOne™ Software v2.0 (ABI, Carlsbad, CA, USA). Porcine miR-24 and miR-103 were used as an internal control for hypothalamus and pituitary, respectively. All reactions were run in triplicate. The comparative 2^−ΔΔ^*^Ct^* method was used to determine the expression level differences.

As Li *et al.* [17] suggested, the expression level at each stage were compared to D60, and the raw real-time PCR and microarray data were scaled to the fold changes. The Pearson correlation coefficient was used to determine the consistency between real-time PCR and microarray data.

### 4.6. Target Prediction and Gene Ontology Analysis

TargetScan [44,45] and porcine mRNA database of the NCBI website were used for the target gene prediction. Here context score percentile of 50 was set as the threshold value to filter the prediction results [46]. The gene ontology (GO) analysis of potentially target genes was conducted using the GOrilla tool [47].

## 5. Conclusions

In summary, we detected a total of 175 and 140 porcine miRNAs that were expressed in hypothalamus and pituitary respectively by using Solexa sequencing technology and microarray analysis. 37 and 30 differentially expressed miRNAs representing different expression modes in hypothalamus and pituitary respectively were revealed in this study. Our findings provide miRNA profiles in porcine developing hypothalamus and pituitary, particular in hypothalamus for the first time, and indicate spatial and temporal characteristics of miRNA expressions in porcine brain. Importantly, this inventory of differentially expressed miRNAs profiles in porcine developing hypothalamus and pituitary indicates the potentially target gene regulatory mechanisms in porcine hypothalamus and pituitary, contributing to our understanding of miRNA expression and regulation in hypothalamus and pituitary.

## Figures and Tables

**Table 1:** miRNAs differentially expressed among D60, D120, and D180 (Fold change >2, *q* < 0.001, and FDR < 0.001).

miRNA name	Hypothalamus D120/D60	D180/D120	D180/D60	miRNA name	Pituitary D120/D60	D180/D120	D180/D60
PC-1386-5p-84388	2.46	-	3.14	miR-1343-5p-85950	0.26	14.10	3.73
PC-3p-104049	2.23	-	3.82	miR-149-3p-18074	0.32	13.70	4.39
PC-3p-27494	5.79	-	4.04	PC-3p-81445	0.27	16.94	4.62
PC-3p-32821	3.99	-	3.73	miR-489-3p-7471	-	11.36	14.48
PC-5p-24051	3.11	-	3.18	PC-296-3p-105798	-	9.33	13.00
PC-5p-62644	2.59	-	4.05	PC-296-3p-8261	-	11.22	12.57
miR-149-3p-18074	-	12.20	8.18	PC-5p-105403	-	6.38	8.16
miR-2140-5p-111116	-	6.90	4.52	PC-3p-43527	-	13.93	16.35
miR-2144-3p-85262	-	4.87	4.09	PC-5p-65544	-	24.12	13.78
PC-1386-5p-44207	-	4.08	4.41	ssc-miR-4334	-	13.17	9.34
PC-3p-23978	-	3.00	2.75	PC-3p-23640	0.44	-	0.48
PC-3p-81445	-	21.58	13.83	PN-125b-5p-19840	0.48	-	0.44
PC-5p-54670	-	6.11	3.99	ssc-miR-125b	0.46	-	0.42
PC-5p-58868	-	21.86	12.39	miR-16-5p-20880	0.35	-	0.34
PN-1274a-5p-107057	-	15.13	9.86	ssc-miR-16	0.21	-	0.23
PN-2143-5p-54940	-	6.32	5.06	PN-381-3p-7212	0.28	-	0.29
PN-219-3p-1285	-	4.55	3.21	ssc-miR-127	0.42	-	0.50
PN-494-3p-2497	-	2.72	2.40	miR-7-5p-14	0.11	3.47	0.39
ssc-miR-181a	-	2.75	2.85	PC-7-5p-2349	0.04	9.54	0.36
ssc-miR-181b	-	3.08	3.20	ssc-miR-7	0.03	6.98	0.21
PC-3p-12824	0.34	7.86	2.65	miR-29a-3p-51	0.40	3.20	-
PC-3p-71633	0.36	8.03	2.90	ssc-miR-29a	0.39	3.13	-
PC-5p-16648	0.19	14.62	2.85	ssc-miR-26a	0.42	2.29	-
PC-720-3p-31375	0.48	4.77	2.28	ssc-miR-125a	0.38	2.30	-
PN-1274b-5p-403	0.19	49.38	9.61	miR-145-5p-335	2.35	-	2.46
ssc-miR-4332	0.21	42.20	8.81	ssc-miR-145	2.65	-	2.43
miR-29a-3p-51	0.43	2.31	-	miR-31-5p-40971	-	0.15	0.20
ssc-miR-127	0.33	2.55	-	PN-2481-5p-34424	-	0.11	0.09
miR-107-3p-19	0.48	-	0.48	ssc-miR-221	-	0.18	0.12
miR-2142-3p-34065	0.46	-	0.46	PN-200a-3p-331	-	0.38	0.30
PN-495-3p-413	0.46	-	0.41				
PN-7-5p-1931	0.10	-	0.07				
ssc-miR-128	0.33	-	0.37				
ssc-miR-191	0.48	-	0.45				
ssc-miR-7	0.07	-	0.08				
PN-125b-5p-19840	-	0.37	0.29				
ssc-miR-125b	-	0.39	0.30				
